# Tissue integration and biodegradation of soft tissue substitutes with and without compression: an experimental study in the rat

**DOI:** 10.1007/s00784-022-04726-0

**Published:** 2022-10-18

**Authors:** Stefan P. Bienz, Cedryck Vaquette, Alexis Ioannidis, Christoph H. F. Hämmerle, Ronald E. Jung, Sašo Ivanovski, Daniel S. Thoma

**Affiliations:** 1 Clinic of Reconstructive Dentistry, University of Zurich, Zurich, Switzerland; 2 School of Dentistry, The University of Queensland, Herston, QLD Australia

**Keywords:** Soft tissue augmentation, Soft tissue substitutes, Histology, Histomorphometry

## Abstract

**Objectives:**

To analyze the influence of compression on tissue integration and degradation of soft tissue substitutes.

**Material and methods:**

Six subcutaneous pouches in twenty-eight rats were prepared and boxes made of Al_2_O_3_ were implanted and used as carriers for soft tissue substitutes: a collagen matrix (MG), two volume-stable collagen matrices (FG/MGA), and a polycaprolactone scaffold(E). The volume-stable materials (FG/MGA/E) were further implanted with a twofold (2) and a fourfold (4) compression, created by the stacking of additional layers of the substitute materials. The samples were retrieved at 1, 2, and 12 weeks (10 groups, 3 time points, *n* = 5 per time point and group, overall, 150 samples). The area fraction of infiltrated fibroblasts and inflammatory cells was evaluated histologically. Due to within-subject comparisons, mixed models were conducted for the primary outcome. The level of significance was set at 5%.

**Results:**

The area fraction of fibroblasts increased in all groups over time. At 12 weeks, the densely compressed materials FG4 (1.1%), MGA4 (1.7%), and MGA2 (2.5%) obtained lower values as compared to the other groups, ranging between 4.7 (E2) and 6.5% (MG). Statistically significant differences (*p* ≤ 0.05) were observed between groups FG4 vs MG/FG2/E/E4 as well as between MGA4 vs MG/FG2/E/E4 and E vs MGA2.

**Conclusions:**

Higher levels of compression led to delayed tissue integration. The effect of different compression levels was more distinct when compared to the differences between the materials.

**Clinical relevance:**

All biomaterials demonstrated tissue integration and a minimal concomitant inflammatory reaction. Clinically, it might be more favorable to obtain a sufficient flap release or to reduce the material size to improve the tissue integration processes.

## Introduction

Surgical interventions aimed at increasing the width of keratinized mucosa or augmenting soft tissue volume around teeth and implants are performed in order to improve biological and esthetic outcomes [1–3]. The techniques for both types of interventions have been described previously and are based on the transplantation of autogenous tissue [4, 5]. However, the harvesting procedures are associated with considerable morbidity for the patient and also with a risk of additional complications [6, 7]. Therefore, soft tissue substitutes have been developed with the aim of replacing autogenous tissue. Soft tissue substitutes offer the advantage of reduced patient morbidity through the avoidance of a second surgical site and should ideally have the potential to reach similar outcomes at the recipient site [8–10].

Volume-stable soft tissue substitutes are being increasingly used for the purpose of augmenting mucosal thickness, particularly in the field of implant dentistry [10, 11]. From a clinical point of view, these soft tissue substitutes should be easy to handle, stable over time, and integrate well with the surrounding tissues. Several volume-stable materials have been developed over the last decade, and many of them were collagen-based [12–15]. Furthermore, polymer-based materials have been applied in related medical fields and might also be suitable for this indication [16–18]. Depending on the structure obtained by different manufacturing techniques, these are designed as a scaffold for bone regeneration [19, 20], soft tissue augmentation, or regeneration of the periodontal ligament [21, 22]. Due to the high processability of polymers, the macro- and microstructure of the resulting scaffold/membrane can be easily tailored depending on the manufacturing technique. While polymeric soft tissue substitutes are still in a developmental phase, they have the potential to be used in a manner similar to that of collagen-based substitutes. So far, the different volume-stable substitute biomaterials (collagen based or synthetic) have not been compared in a systematic manner.

Based on the clinical experience it is speculated that there is an obvious difference between volume substitutes and autogenous tissue in terms of the mechanical behavior with dissimilar resistance to compression. Autogenous tissue grafts are consists of connective tissue and are mainly based on elastic and collagen fibers as well as a fluid that is bound in the extracellular matrix with glycosaminoglycans and proteoglycans [23]. Physically speaking, the structure of an autogenous graft allows only small amounts of compression with pressure from a flap. Conversely, the substitutes are designed like a sponge in order to allow for cells to integrate. Due to their structure, they exhibit low resistance to compression. Presumably, after soft tissue augmentation, these substitutes may experience compression with the closure of the flap. Whether or not this compression has an impact on the biological performance of the substitute materials, including tissue integration and degradation, however, has not yet been investigated.

Therefore, the aim of the present preclinical study was (i) to test whether or not the compression of volume-stable substitutes affects tissue integration and degradation and (ii) whether the integration and degradation differ between different soft tissue substitutes.

## Material and methods

### Study design

This study was designed as an experimental, randomized-controlled trial. A total of 28 adult female rats (Sprague Dawley, Rattus Norvegicus) were used in the present study. Study design, animal selection, housing, and surgery protocols were approved by the local Animal Ethics Unit, Office of Research Ethics, The University of Queensland, Australia (DENT/434/19). The animals were bred at the Animal Resource Centre in Western Australia. From 1 week before the commencement of the trial until sacrifice, the animals were housed in a laboratory with a physical containment level of 2 (PC2). The rats were group housed with a maximum of three animals per cage. Single housing was conducted during the first 7 days following surgery. Cages were equipped with wood chip bedding and ad libitum access to rat pellets and water.

### Production of boxes made of alumina

**Fig. 1:**
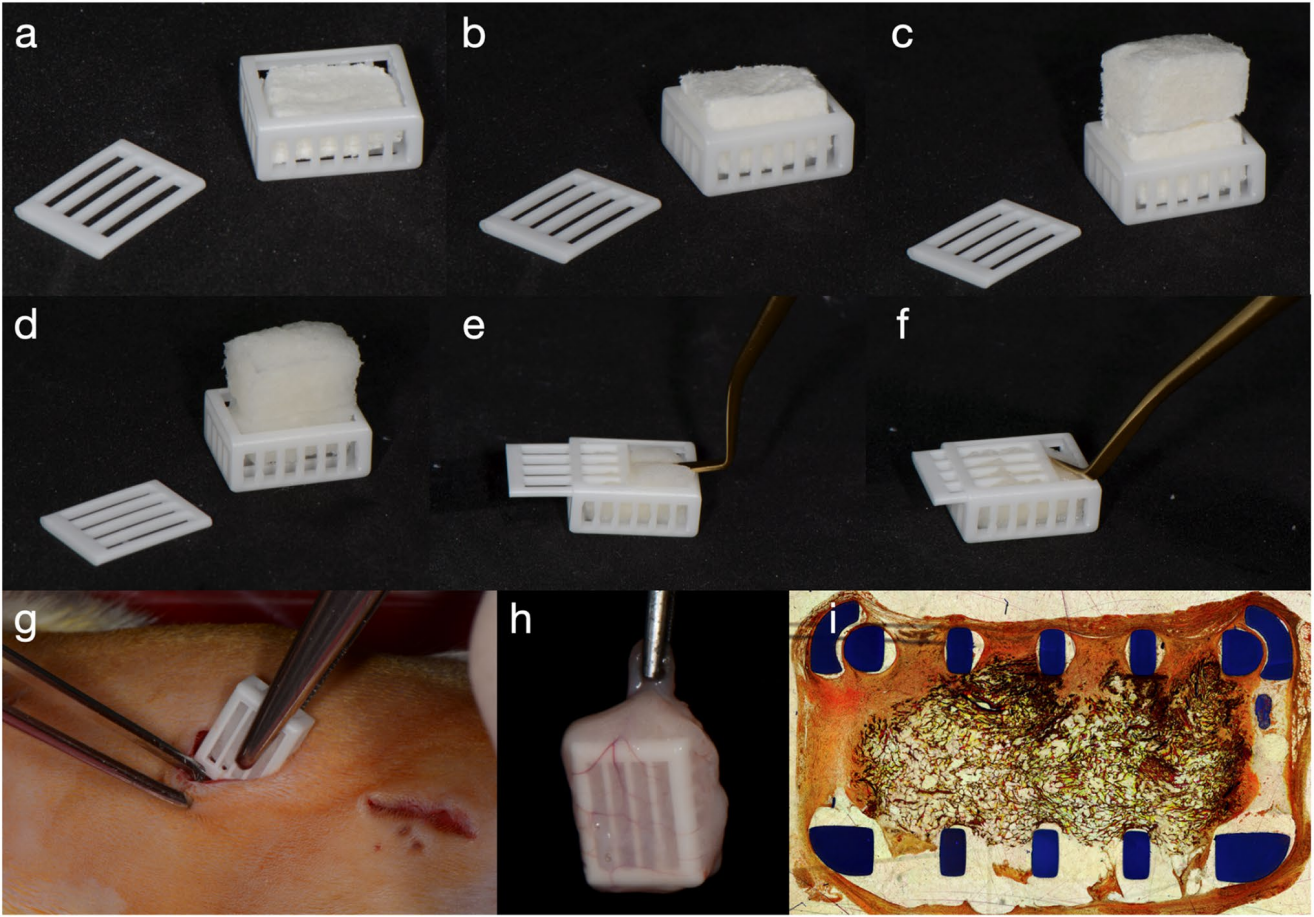
Box with slide cover, printed in densely sintered alumina. The inner dimensions of 10 mm by 7.5 mm by 3 mm were selected in order to facilitate the substitute preparation. In this way, most materials could be cut in half based on their original dimensions. Representative pictures of the loading of the boxes with a substitute of **a** 3 mm thickness (FG), **b** 6 mm thickness (FG2), and **c** 12 mm thickness (FG4). The materials (here FG4) were then **d** soaked with saline and then **e** compressed in order to **f** close the slide cover. The box was then **g** placed in a subcutaneous pouch and **h** removed at the specific time-point (here 1 week of healing). A histologic slide **i** of the box is shown, produced with methylmethacrylate embedding and microcutting and grinding technique. This is a representative picture; the samples used for analysis were embedded in paraffin, without the box

In order to standardize the volume of the substitutes and to further simulate the compression of the volume-stable materials, boxes made of Al_2_O_3_ were specifically designed in order to feature regularly spaced apertures, enabling tissue infiltration (Fig. 1–1). Al_2_O_3_ shows comparable biocompatibility as ZrO_2_ in terms of cell viability [24] but facilitated the printing process. The inner dimensions of the boxes were 10 mm by 7.5 mm by 3 mm. The box was closed by sliding a porous lid. Three-dimensional printing of high-performance alumina was conducted by using lithography-based ceramic manufacturing (LCM) process on a CeraFab 7500 Dental 3D printer (Lithoz GmbH, Vienna, Austria). The LCM process uses light-curable suspensions of ceramic particles in organic resin systems as starting materials. This liquid starting material was cured with blue light (λ = 465 nm) by means of photopolymerization in a DLP-like 3D printing process, thus leading to a solid three-dimensional green body. Subsequently, uncured material was removed using the commercial cleaning fluid LithaSol 20 in an airbrush cleaning station (CeraClean Ultra, Lithoz GmbH). Thereafter, thermal post-processing led to fully dense, pure ceramic bodies. Debinding (i.e., thermal removal of the polymeric binder) was achieved by heating the green bodies up to a temperature of 1100 °C followed by sintering at 1600 °C with a dwell time of 2 h. A theoretical density of 99.4% of the theory and a four-point bending strength of 430 MPa were achieved with this process [25]. The resulting material, LithaLox HP 500 (Lithoz GmbH, Vienna, Austria), features a high purity (99.99% aluminum oxide) and low surface roughness (*R*_a_ ≈ 0.4 µm).

### Study materials and group allocation

The study consisted of 4 different materials, divided into 10 groups.

- MG (Geistlich Mucograft®, Geistlich Pharma AG, Wolhusen, Switzerland) served as reference material. Due to the lack of volume stability, MG was used as a single layer and was not compressed.
- FG (Geistlich Fibro-Gide®, Geistlich Pharma AG) was evaluated with a thickness of 3 mm (according to the dimension of the box), a thickness of 6 mm (FG2), and a thickness of 12 mm (FG4) (Fig. 1a–c).
- In a similar way, MGA (creos^tm^ mucogain, Nobel Biocare AB, Göteborg, Sweden) was prepared with a thickness of 3 mm, 6 mm (MGA2), and 12 mm (MGA4).
- A melt electrowritten scaffold made of medical grade polycaprolactone (PC12, Corbion) was manufactured at the University of Queensland via a direct melt electrowriting approach previously described [26, 27], whereby a programmable *x*–*y* stage is used to collect the fibers. The polymer was melted electrospun through a blunt 21G needle using an extrusion pressure of 0.7 bar, a voltage of 7 kV, and a spinneret collector distance of 8 mm. The temperatures of heater #1 (placed near the syringe) and heater #2 (placed near the needle) were set to 75 and 85 °C, respectively. The translational speed of the collector was set at 450 mm/min in order to obtain straight fibers, and a square wave pattern was utilized for fabricating a scaffold composed of alternating series of layers oriented at 90° where the fiber interdistance was 400 µm. The scaffolds were produced in a thickness of 1.5 mm and were then stacked in order to reach 3 mm (E), 6 mm (E2), and 12 mm (E4).

A class 2 biosafety cabinet in the surgery room was used in order to precut and soak the materials with saline, prior to placing them in the boxes (Fig. 1d–f), which were subsequently closed with the lid.

### Primary endpoint, sample size, pilot study, number of animals, and randomization

The primary endpoint was defined as the area fraction of fibroblasts at 12 weeks of healing. The area fraction of fibroblasts was assessed as a percentage within the region of interest (see *histomorphometric analysis*). Power calculation was done for independent groups and continuous variables. For an expected 6% difference between the means of two groups with a standard deviation of 2%, a sample size of 4 gave a statistical power of 80%. Due to modifications of the animal model with the newly used boxes, the number per subgroup was raised to 5. Power calculation was performed using an online tool (https://clincalc.com/stats/samplesize.aspx).

The influence of the boxes on the integration process was unclear, as they blocked roughly 75% of the outer surface of the substitute. Therefore, a pilot study was conducted in order to verify the results at 1 week. It appeared that at 1 week, there was a very limited number of cells available in the region of interest. This was considered ideal as an early time-point. The time points of evaluation were then defined as 1 week, 2 weeks, and 12 weeks, and the results of the pilot study were included in the analysis. Ten groups with *n* = 5 and 3 evaluation time-points resulted in 150 specimens overall. A computer-generated list was prepared for the allocation of all specimens (https://www.random.org/) and the animals carried up to 6 boxes each.

### Surgery

Anesthesia was performed using a Mediquip Isoflurane vaporizer. Induction was performed at 4% isoflurane and anesthesia maintenance was performed at 2%. The animal revival was then assisted using pure oxygen. The rats were kept on a heating pad during surgery (approx. 25 min) and postoperatively to maintain body temperature. Preemptive multi-modal analgesia (buprenorphine 0.01–0.05 mg/kg and meloxicam 1 mg/kg) was provided, as well as postoperative analgesia with tramadol mixed with drinking water (7 days post-op). Preemptive prophylaxis was provided by subcutaneously administering Kefzol (20 mg/kg) and Gentamicin (5 mg/kg). Rats were depilated in the dorsal area with an electric shaver. Following disinfection with povidone-iodine (Betadine®, Mundipharma, Basel, Switzerland), three paramedian incisions of 15 mm length were made along and on each side of the vertebral column. A subcutaneous pouch was created using blunt scissors before the loaded boxes were inserted into the pouch (Fig. 1g). The incision was then closed by using 3–4 surgical staples (size 7). The animals were monitored daily during the first 7 days and 3 times a week thereafter. Following the healing period, the animals were euthanized by CO_2_ (a fill rate of 10–30% of the chamber volume per minute with CO_2_, added to the existing air in the chamber). Specimens were retrieved (Fig. 1h), removed from the boxes, and immediately stored in paraformaldehyde. One additional sample (in the animal carrying only 2 boxes for analysis) of each evaluation time point was fixed together with the box for descriptive purposes.

### Histology

After 48 h of fixation in paraformaldehyde, specimens were stored in phosphate-buffered saline. Following a maximum of 5 days of storage, specimens were dehydrated and infiltrated with xylol and paraffin (paraffin at 60 °C). The central area was cut into 2- to 5-μm-thick sections with a microtome (MICROM, Medite GmbH, Dietlikon, Switzerland). One suitable section was stained with hematoxylin–eosin.

The samples obtained together with the box were dehydrated and infiltrated with xylol before embedding in polymethylmethacrylate. One central cross-section was prepared by a microcutting and grinding technique (approximately 60 μm thickness, Van Gieson–Elastica staining; Fig. 1i) [28]. For each material, one pristine sample was dehydrated, embedded in polymethylmethacrylate, and also prepared with the microcutting and grinding technique.

### Histomorphometric analysis

**Fig. 2:**
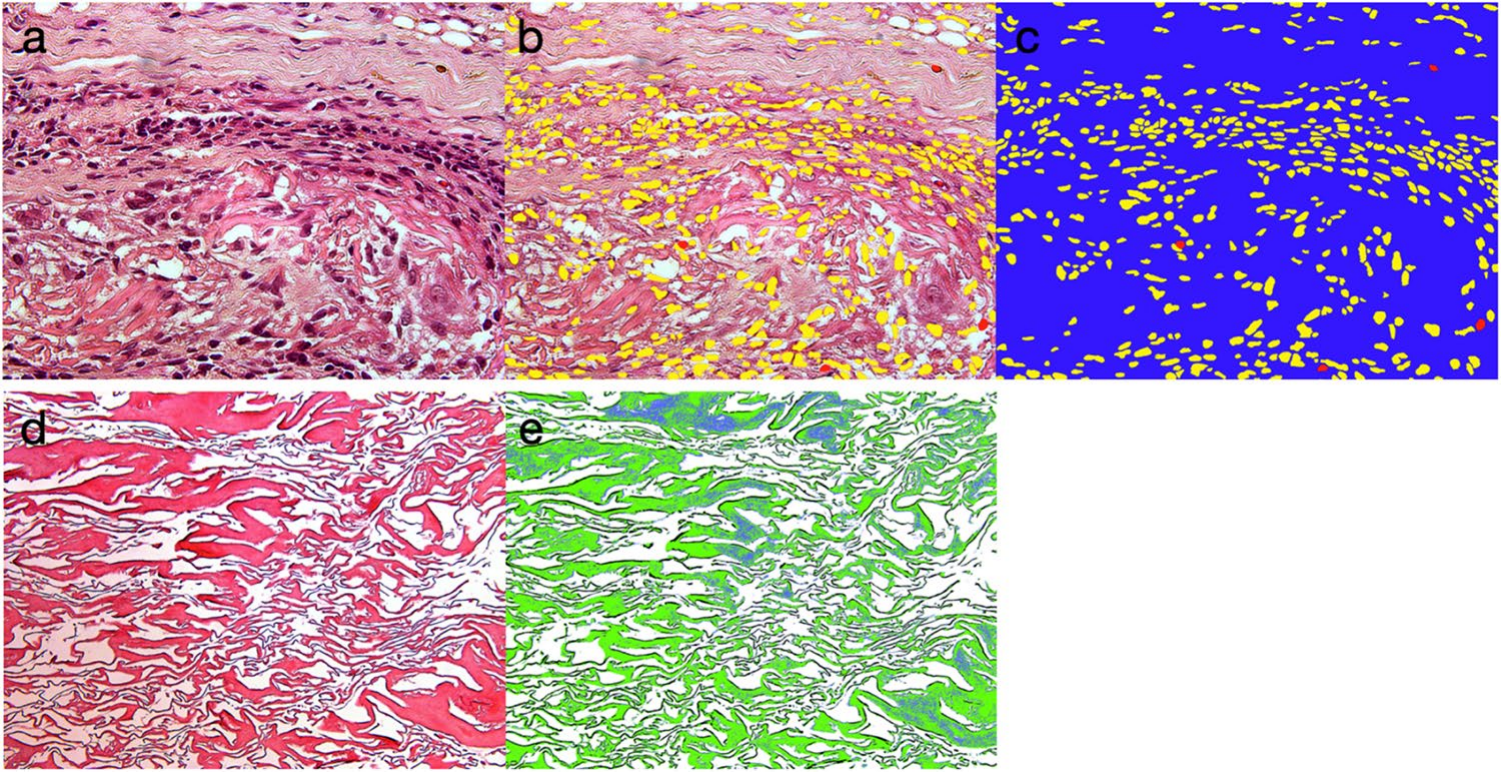
For the histomorphometric evaluation of cells, a × 500 magnification in the center of the slide/box (**a**) was used. Inflammatory cells (red) and fibroblasts (yellow) were marked (**b**) and the remaining area (**c**) was considered as background (blue). The area fraction was derived as a percentage of the overall region of interest. For the histomorphometric evaluation of connective tissue and residual substitute, a × 200 magnification in the center of the slide/box (**d**) was used. In the present illustration, the residual substitute was marked by posterization (**e**) and the amount was again derived as a percentage of the overall region of interest

The analysis was performed by a blinded histologist using an optical microscope (Leica DM6000 B, Leica, Wetzlar, Germany) equipped with a digital camera (Leica DFC 450, Leica). One region of interest, standardized in size, was located in the center of the specimen at a × 50 magnification. In the case of group MG, the region of interest was located in the dense layer of the substitute. An image editing software (Adobe Photoshop CS6 Extended, Adobe Systems, San José, CA, USA) was used to mark inflammatory cells, fibroblasts, and background (background being mainly the extracellular matrix) (Fig. 2a–c). After the cells were marked with a color, the percentage of the area of that particular color in relation to the overall area of the region of interest was calculated (LAS V4.3, Leica). This is considered a semi-quantitative analysis, therefore, in contrast to quantitative analyses, which are most often counting cells.

A second digital picture was taken at a × 20 magnification, also located in the center of the section. The same image editing software was used to mark connective tissue and the remaining substitute by means of separating them according to their different shades (tool: “posterization,” Adobe Photoshop CS6 Extended) (Fig. 2d, e). Depending on the image, 3–7 color levels were used to adjust for connective tissue and substitute recognition. The percentage of the area of all subgroups was calculated in a similar way for a semi-quantitative analysis (LAS V4.3, Leica). Based on the same image with × 20 magnification, the number of blood vessels was counted (Adobe Photoshop CS6 Extended).

The sections containing the pristine materials (not implanted) were assessed similarly by using posterization at a × 20 magnification. The material as well as the background were assessed in order to estimate the porosity of the materials.

### Statistical analysis

Data was computed in a spreadsheet (Excel, Microsoft Corp., Redmond, WA, USA) and was then imported into statistical analysis software (SAS Corp., NC, USA). A ratio of the area fraction of inflammatory cells in relation to the area fraction of fibroblasts was calculated. Means and standard deviations, as well as medians with quartiles, were used to describe continuous variables, and counts and percentages were used for categorical variables. Due to within-subject comparisons and unclear data distribution, parametric and nonparametric mixed models were conducted for the primary outcome, including a Bonferroni adjustment. The level of significance was set at 5%. Scatterplots were produced with statistical analysis and graphics software (Prism, version 8.2.0, GraphPad Software Inc., Playa La Jolla, CA, USA).

## Results

### Descriptive findings

Postoperative monitoring reported no complications throughout the entire observation period. The soft tissue integration of the boxes was clinically favorable, with intimate soft tissue contact and no signs of redness or edema. Blood vessels surrounding and entering the box were visible around the boxes at 1 week (Fig. 1h). All specimens were retrieved successfully and underwent tissue processing for histology. Connective tissue, number of blood vessels, and substitute degradation were not evaluated in 5 specimens due to the insufficient quality of the histologic slides. The sections embedded in polymethylmethacrylate revealed early connective tissue formation in between and around the bars of the box. The tissue revealed good adherence to the box material and early integration of the matrix was visible already at 1 week (Fig. 1i).

### Porosity of pristine materials

The porosity of the pristine materials in a pre-wettened state, without compression, amounted to 60.76% for MG (dense layer), 81.88% for FG, 57.85% for MGA, and 98.30% for E.

**Fig. 3:**
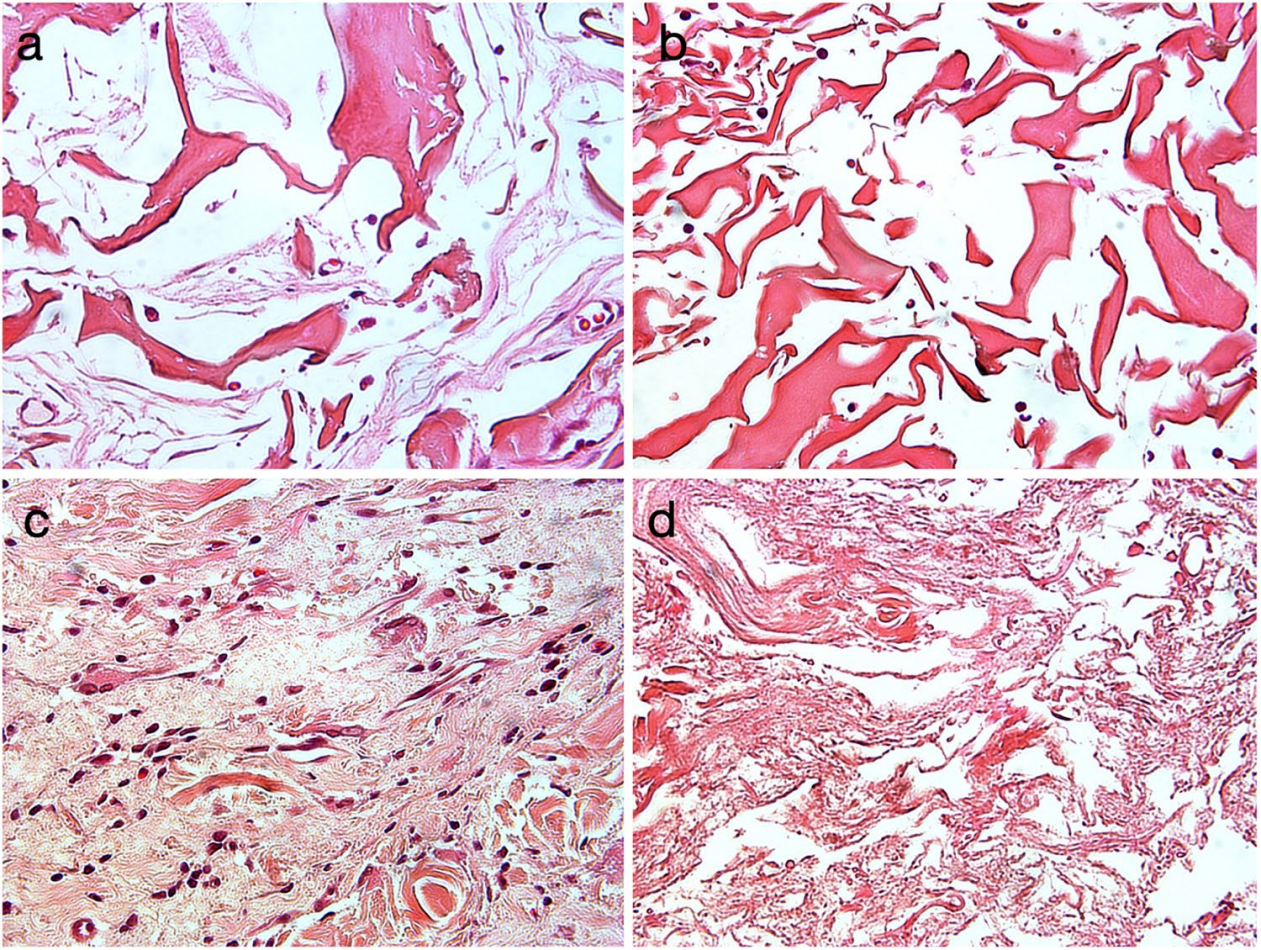
All pictures present histological slides at 12 weeks: **a** FG = volume-stable collagen matrix (Geistlich Fibro-Gide®), **b** FG4 same material with fourfold compression, **c** MGA = volume-stable collagen matrix (creos^tm^ mucogain), and **d** MGA4 same material with fourfold compression

All descriptive and quantitative results are depicted in Fig. 3 and descriptive data are summarized in Appendix Table 1.

### Fibroblasts

**Fig. 4:**
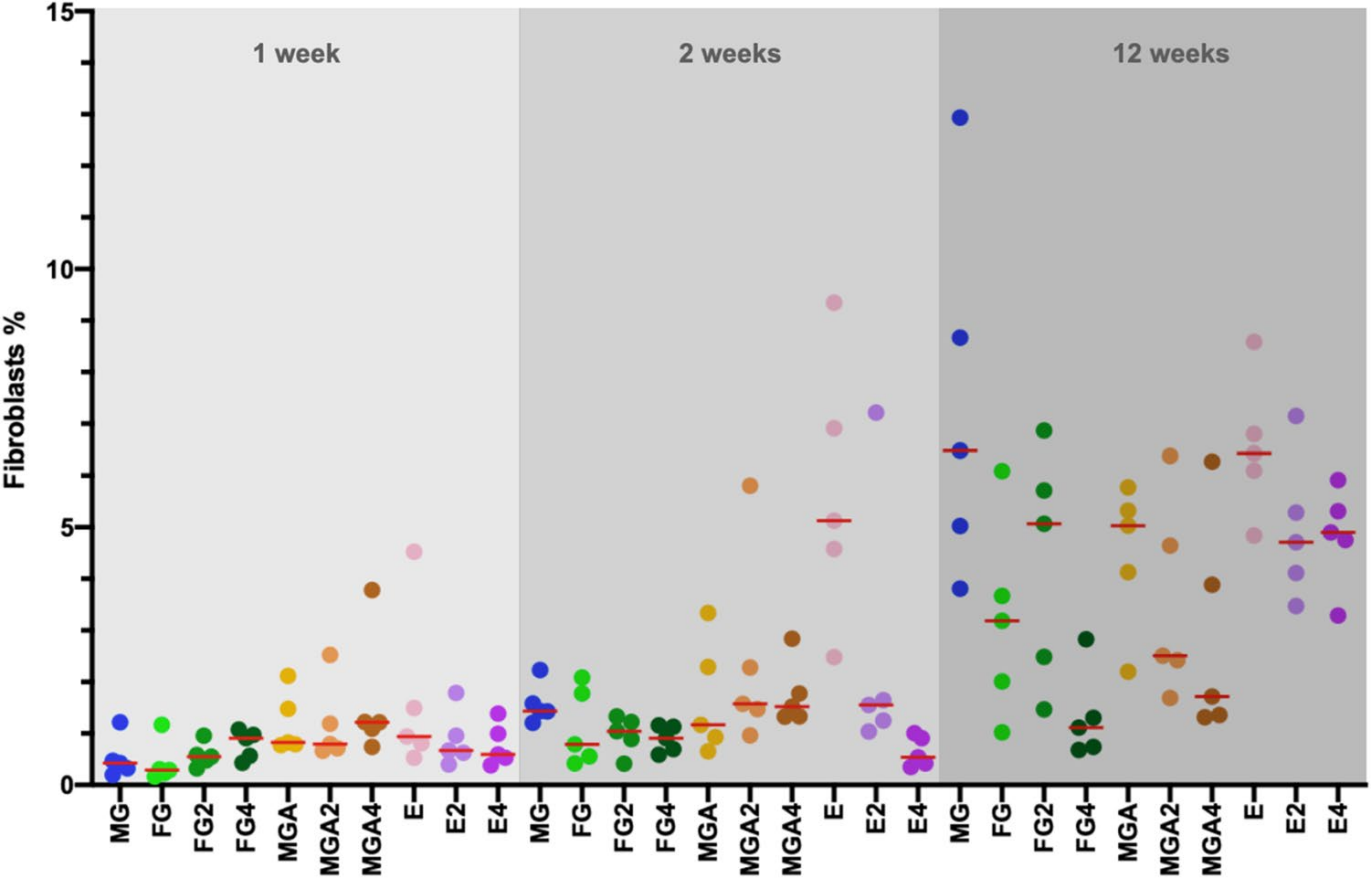
Scatterplot depicting the amount of fibroblasts. Red lines represent the median. MG = collagen matrix (Geistlich Mucograft®); FG = volume-stable collagen matrix (Geistlich Fibro-Gide®) with twofold compression (FG2) and fourfold compression (FG4); MGA = volume-stable collagen matrix (creos^tm^ mucogain) with twofold compression (MGA2) and fourfold compression (MGA4); E = scaffold made of polycaprolactone (PCL-12) with twofold compression (E2) and fourfold compression (E4)

The median area fraction of fibroblasts at 1 week was ranging from 0.29 (FG; minimum: 0.16; maximum: 1.17) to 1.22% (MGA4; min: 0.74; max: 3.78) (Fig. 4). From 1 to 2 weeks, the area fraction of fibroblasts slightly increased in most groups and ranged between 0.54 (E4; min: 0.35; max: 1.01) and 1.57% (MGA2; min: 0.96; max: 5.81), with the exception of 5.13% (E; min: 2.48; max: 9.34).

Up to 12 weeks, the area fraction of fibroblasts increased in all groups. Compression in groups FG and MGA had a significant influence on the outcome, with groups FG4 (1.11%, min: 0.68; max: 2.83), MGA4 (1.72%; min: 1.32; max: 6.27), and MGA2 (2.51%; min: 1.69; max: 6.38) obtaining lower values as compared to the other groups, which all ranged between 3.18% (FG, min: 1.02; max: 6.09) and 6.48% (MG; min: 3.81; max: 12.94). This is also represented as an example in Fig. 3a–d, showing a comparison of FG vs FG4 and MGA vs MGA4 at 12 weeks. Parametric mixed model analyses revealed statistically significant differences (*p* ≤ 0.05) between groups FG4 vs MG/FG2/E/E4 as well as between MGA4 vs MG/FG2/E/E4 and E vs MGA2. With nonparametric mixed models, MGA4 vs FG2/E4 was not statistically significant, but FG4 vs MGA reached statistical significance (*p* ≤ 0.05).

### Inflammatory cells

**Fig. 5:**
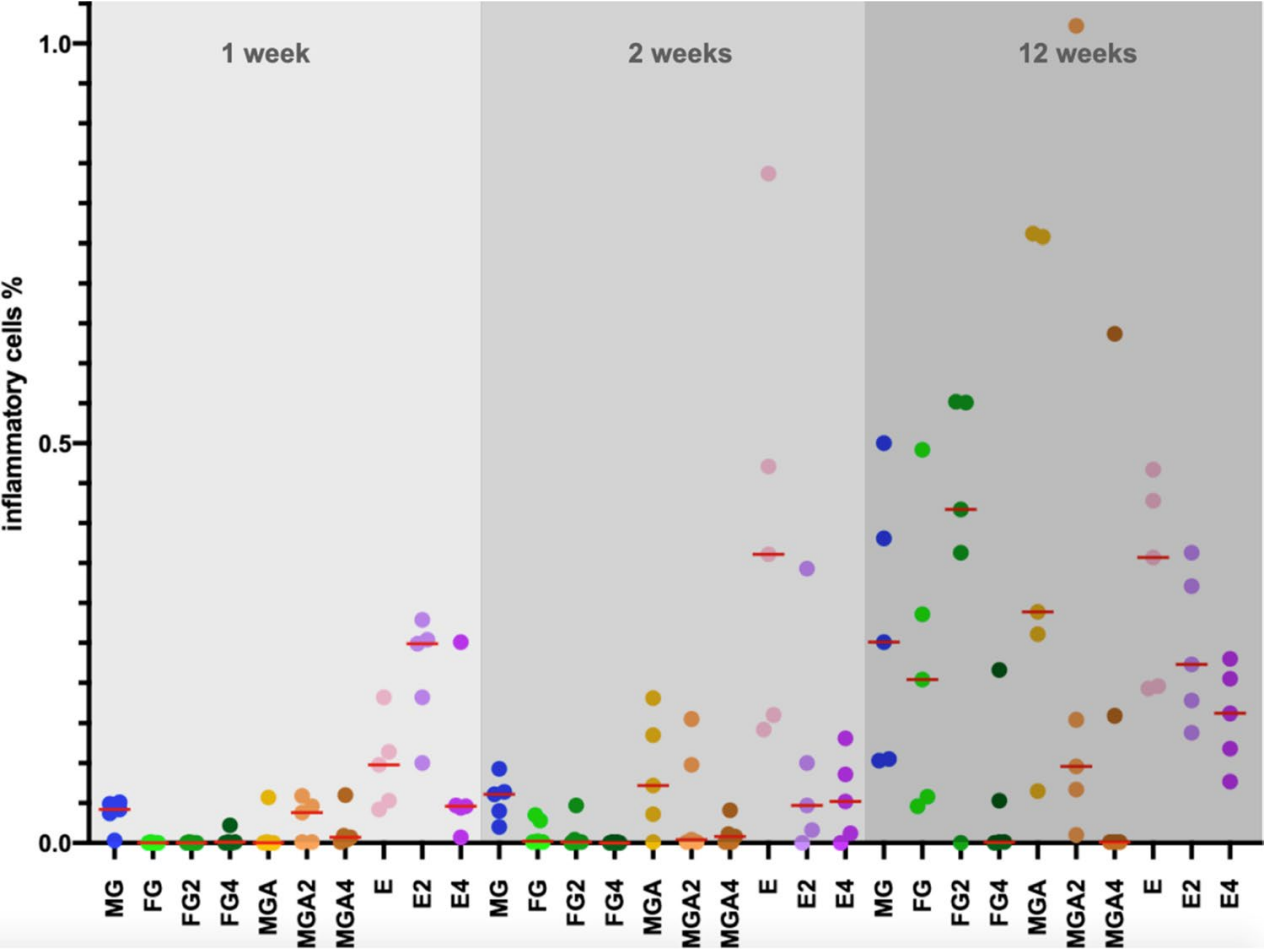
Scatterplot depicting the amount of inflammatory cells. Red lines represent the median. MG = collagen matrix (Geistlich Mucograft®); FG = volume-stable collagen matrix (Geistlich Fibro-Gide®) with twofold compression (FG2) and fourfold compression (FG4); MGA = volume-stable collagen matrix (creos^tm^ mucogain) with twofold compression (MGA2) and fourfold compression (MGA4); E = scaffold made of polycaprolactone (PCL-12) with twofold compression (E2) and fourfold compression (E4)

The median area fraction of inflammatory cells was higher in group E2 (0.25%; min: 0.10; max: 0.28) at 1 week as compared to the other groups, ranging from 0 (FG, FG4; min: 0; max: 0) to 0.10% (E; min: 0.04; max: 0.18) (Fig. 5). At 2 weeks, group E (0.36%; min: 0.14; max: 0.84) showed an increased amount as compared to the other groups, ranging from 0 (FG; min: 0; max: 0) to 0.07% (MGA; min: 0.00; max: 0.18).

**Fig. 6:**
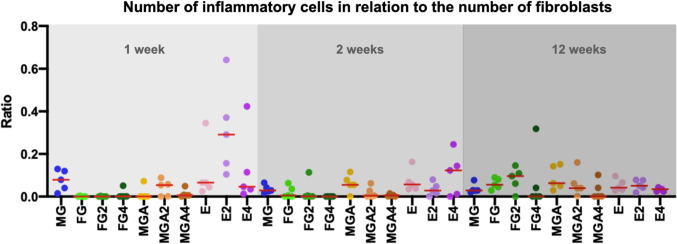
Scatterplot depicting the amount of inflammatory cells in relation to the amount of fibroblasts. Red lines represent the median. MG = collagen matrix (Geistlich Mucograft®); FG = volume-stable collagen matrix (Geistlich Fibro-Gide®) with twofold compression (FG2) and fourfold compression (FG4); MGA = volume-stable collagen matrix (creos^tm^ mucogain) with twofold compression (MGA2) and fourfold compression (MGA4); E = scaffold made of polycaprolactone (PCL-12) with twofold compression (E2) and fourfold compression (E4)

An increase occurred for most groups up to 12 weeks. Low amounts of inflammatory cells at 12 weeks were obtained for FG4 (0.00%; min: 0.00; max: 0.22) and MGA4 (0.00%; min: 0.00; max: 0.64). In relation to the area fraction of fibroblasts, the area fraction of inflammatory cells did not clearly change over time and was very homogenous comparing all groups (Fig. 6).

### Connective tissue formation and vascularization

**Fig. 7:**
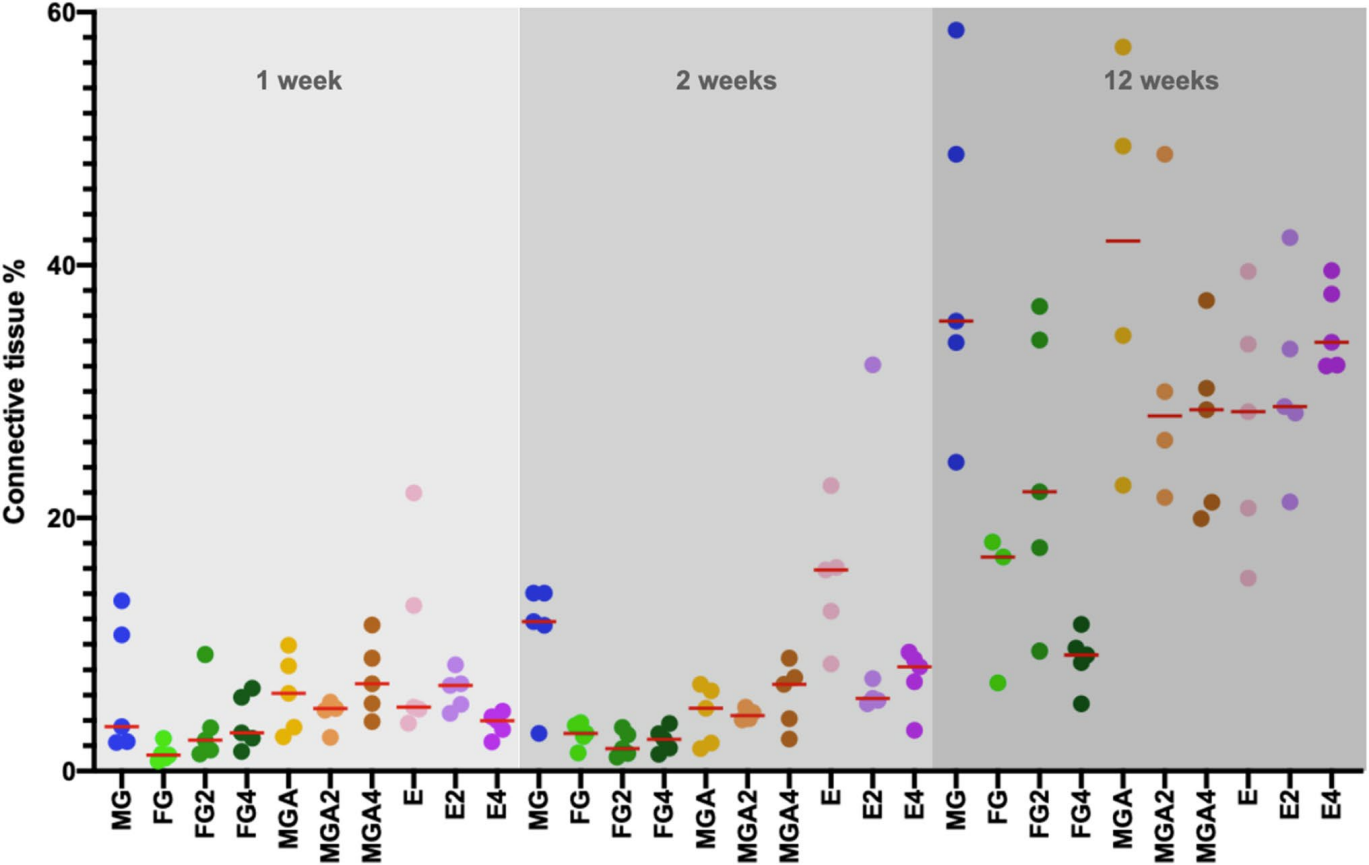
Scatterplot depicting the amount of newly formed connective tissue. Red lines represent the median. MG = collagen matrix (Geistlich Mucograft®); FG = volume-stable collagen matrix (Geistlich Fibro-Gide®) with twofold compression (FG2) and fourfold compression (FG4); MGA = volume-stable collagen matrix (creos^tm^ mucogain) with twofold compression (MGA2) and fourfold compression (MGA4); E = scaffold made of polycaprolactone (PCL-12) with twofold compression (E2) and fourfold compression (E4)

**Fig. 8:**
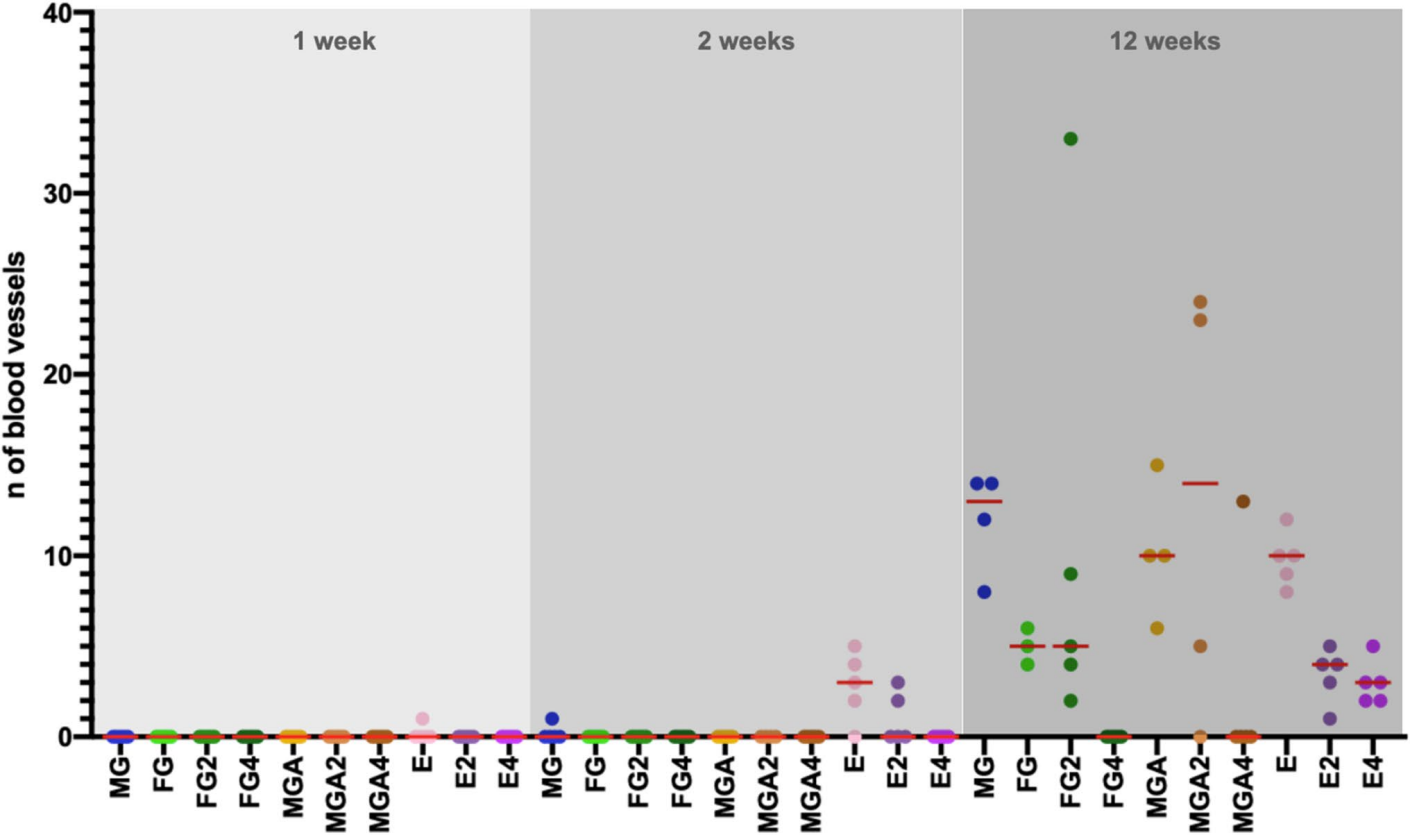
Scatterplot depicting the number of blood vessels. Red lines represent the median. MG = collagen matrix (Geistlich Mucograft®); FG = volume-stable collagen matrix (Geistlich Fibro-Gide®) with twofold compression (FG2) and fourfold compression (FG4); MGA = volume-stable collagen matrix (creos^tm^ mucogain) with twofold compression (MGA2) and fourfold compression (MGA4); E = scaffold made of polycaprolactone (PCL-12) with twofold compression (E2) and fourfold compression (E4)

Details on the connective formation and the number of blood vessels are available in Figs. 7 and 8 as well as in Appendix Table 1. In general, the area fraction of connective tissue followed the trends of the area fraction of fibroblasts as well as the formation of blood vessels, with an increased number at 12 weeks.

### Matrix degradation

**Fig. 9:**
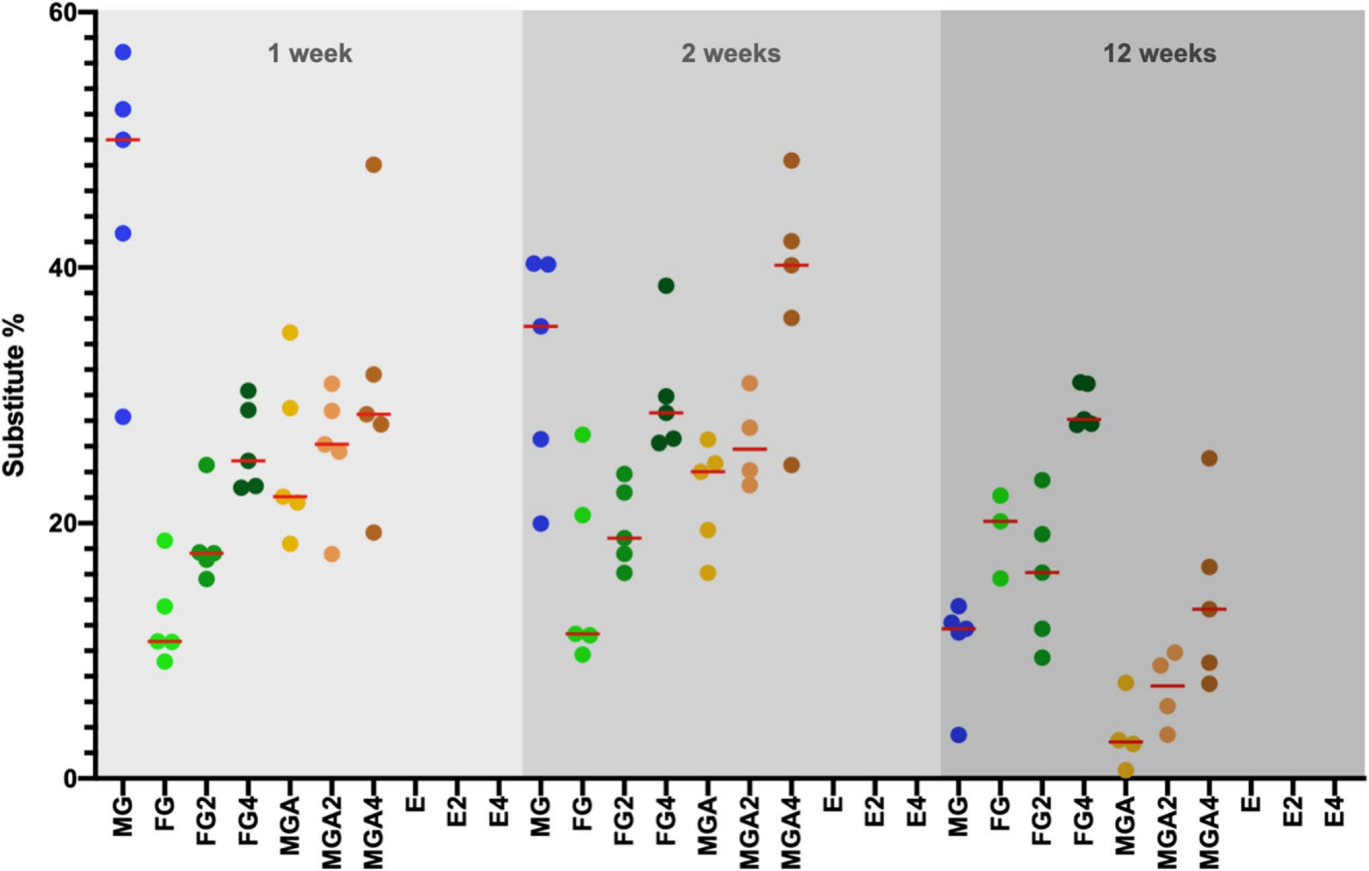
Scatterplot depicting the residual substitute. Group E could not be evaluated with the present histological technique. Red lines represent the median. MG = collagen matrix (Geistlich Mucograft®); FG = volume-stable collagen matrix (Geistlich Fibro-Gide®) with twofold compression (FG2) and fourfold compression (FG4); MGA = volume-stable collagen matrix (creos^tm^ mucogain) with twofold compression (MGA2) and fourfold compression (MGA4); E = scaffold made of polycaprolactone (PCL-12) with twofold compression (E2) and fourfold compression (E4).

The highest amount of residual substitute at 1 week was obtained in group MG (50.00%; min: 28.32; max: 56.88), followed by a reduction to 11.74% (min: 3.41; max: 13.51) at 12 weeks (Fig. 9). Group MGA amounted to 22.07% (min: 18.40; max: 34.93) at 1 week and decreased to 2.86% (min: 0.65; max: 7.49) at 12 weeks. MGA2 and MGA4 decreased over time as well, with slightly higher values at all time points (MGA2: 26.17 to 7.26%; MGA4: 28.50 to 13.26%). Group FG had the lowest value of all groups at 1 week (10.75%; min: 9.17; max: 18.65). Similar to the MGA groups, the compressed groups of FG had higher amounts of residual substitute at 1 week (FG2: 17.64%; FG4: 24.89%). However, the material remained stable over 12 weeks (FG: 20.16%; FG2: 16.14%; FG4: 28.12%). The amount of remaining PCL in groups E, E2, and E4 was not assessed with the present methodology due to the micrometric features of the electrospun fiber. However, a minimal amount of degradation is expected to occur within the 12-week experimental time frame as PCL requires several years to degrade [29].

## Discussion

The present trial predominantly revealed: (i) a progressive tissue integration over time for all materials and a concomitant inflammatory response; (ii) a delayed tissue integration for densely compressed FG and MGA; (iii) continuous degradation of MG and MGA over 12 weeks.

The lower resistance of substitutes against compression forces as compared to connective tissue grafts was considered the main difference following the first years of clinical experience with volume-stable substitutes [10, 11]. All substitutes are porous and elastic, and it is, therefore speculated that they are prone to compression. In the present study, a high level of compression resulting in the densification of the tissue substitute impaired the integration of FG and MGA (Fig. 3a–d). This was indicated by a reduced presence of fibroblasts at 12 weeks and a lack of blood vessels as well as a trend toward less connective tissue ingrowth. An increased amount of substitute material was used in the same volume, thereby lowering the porosity. This is also supported by the higher percentage of residual substitutes found in the highly compressed groups. Interestingly, the integration of the melt electrowritten substitute did not seem to be affected by the compression, at least in the late time point (12 weeks). This might be explained by the higher porosity of the electrospun scaffold enabling tissue infiltration regardless of the level of compression.

The difference in tissue infiltration is also affected by the micro-architectural features of the substitutes. FG consists of a loose 3D structure without a distinct orientation of channels and has a varying pore size [30]. MGA consists of pore sizes of 100 µm and parallelly orientated channels [13]. In contrast, the melt electrowritten scaffold is composed of highly ordered micron-scaled fibers, thereby creating an interconnected network of macropores (400 µm in size). It remains unclear whether the microstructure had an influence on the particular results of this trial, however other studies have reported the influence of pore sizes for related fields [31]. Overall, these findings indicate that under limited compression, a favorable tissue integration of all substitutes can be expected, consistent with other reports [13, 32, 33] (Figs. 4, 5, and 6).

Another requirement that soft tissue substitutes need to fulfill is biocompatibility so that they only trigger a limited inflammatory response. In general, all substitutes elicited a similar and low inflammatory response at early time points except for the melt electrowritten scaffold, possibly due to the synthetic polymer nature being prone to inducing an initial mild foreign body response [34]. Previous studies revealed that topographical changes can affect the cellular adhesion, migration, and production of inflammatory cytokines [35, 36]. The weak initial inflammatory response in FG and MGA might be explained by an alteration of the intrinsic topographical characteristics of the substitute materials. Interestingly, the level of inflammation decreased in the melt electrowritten groups at 12 weeks, suggesting a resolution of the inflammatory process.

The degradation of substitutes is an important parameter since an early degradation may not meet the needs for soft tissue augmentation over the long term. All materials exhibited different degradation rates over time. This finding is most likely explained by their material composition. MG displayed a high degradation rate, most likely attributed to its structure of native collagen without cross-linking [37]. Cross-linking is a physical, chemical, or physicochemical process that enhances the stability of collagen counteracting its rapid degradation [38, 39]. Consequently, the cross-linked FG showed the lowest resorption rate, or almost no resorption over the investigated 12 weeks. Other preclinical and clinical studies reported that FG was also clearly visible at 90 days, although quantification of residual FG was not performed [10, 40]. However, the finding is in contrast to an earlier report with a distinct degradation profile over time [30], although comparisons are difficult as the histologic methodology was different and the effect of the box remains unknown. Observation periods exceeding 3 months might be required in order to more thoroughly understand the behavior of the material. In addition, a decreasing rate of degradation was found under increasing compression, but this merely reflects the initial higher amounts of material, which obviously requires more time for degradation. It also indicated that the impediment of tissue colonization delays the subsequent remodeling of the collagen-based substitutes [38].

The clinical implications of the present findings remain, to some extent, a matter of speculation. All tested materials have shown good tissue integration without signs of increased inflammatory reactions. While a limited compression did not result in obvious changes to the properties of the substitutes, a high level of compression greatly affected tissue colonization of the substitutes. One may argue that a fourfold compression is not a clinically realistic scenario. However, according to clinical experience, a non-compressed status of the substitute cannot be achieved underneath a flap [41]. Therefore, groups FG2, MGA2, and E2 are considered to reflect the usual clinical environment. A dense, fourfold compression is expected in case of increased flap tension or locally above prominent bony profiles such as the buccal crest, i.e., the area where a gain in soft tissue volume is mostly needed [42, 43].

Application, integration, and handling of the boxes proved to be safe and predictable and more importantly, enabled the application of compression in a controlled and reliable manner, which is not easily otherwise possible in a subcutaneous model. Therefore, the present method offered further advantages over other preclinical studies. Earlier studies placed substitutes in a subcutaneous pouch and usually marked the specimen with a suture [30, 38, 44]. However, sutures can cause additional inflammatory side effects [45], therefore potentially biasing the findings. Furthermore, at removal, especially at later time points, identification of the specimen remained difficult. Consequently, the region of the histological slide was defined arbitrarily, as was the location of the region of interest. In contrast, the box used in the present study enabled the removal of a defined block of tissue. This again allowed a reproducible positioning of the region of interest, along the long axis of the box and in the center, thereby ensuring that all samples were evaluated in the same location and hence validating their comparison from this standpoint.

In contrast to the above-mentioned advantages, a limitation of the study is that it remains unclear to what extent the box limited cellular migration and the biomaterial-host events. The use of a box also complicates the comparison with other studies. Ideally, research would benefit from this model and improve standardization when used repeatedly. Another limitation is that the histological analysis did not include immunohistochemistry or gene expression for a more thorough understanding of the healing process.

Future research should more thoroughly investigate the mechanical properties of the substitute materials in comparison with the connective tissue graft. Furthermore, a clinical setting might be able to more thoroughly investigate the relationship between integration and the gain in volume.

## Conclusion

Tissue integration and level of inflammation were comparable for collagen-based, volume-stable collagen-based, and volume-stable polycaprolactone-based scaffolds. For volume-stable materials, high levels of compression led to a delayed integration and the ensuing inflammatory response but a similar compression-dependent degradation. These preclinical results indicate that the integration of substitute materials is delayed in clinical scenarios with insufficient flap release or an excessive amount of substitute material.

## Appendix

**Table 1:** All descriptive results are summarized in the present table

Group	Time	Variable	*N*	Mean	SD	Min	Q1	Median	Q3	Max
MG	1	Fibroblasts	5	0.53	0.40	0.20	0.32	0.43	0.47	1.22
MG	2	Fibroblasts	5	1.58	0.39	1.21	1.43	1.44	1.58	2.23
MG	12	Fibroblasts	5	7.39	3.59	3.81	5.02	6.48	8.67	12.94
FG	1	Fibroblasts	5	0.43	0.42	0.16	0.23	0.29	0.31	1.17
FG	2	Fibroblasts	5	1.13	0.76	0.42	0.56	0.79	1.78	2.08
FG	12	Fibroblasts	5	3.19	1.92	1.02	2.01	3.18	3.67	6.09
FG2	1	Fibroblasts	5	0.58	0.23	0.32	0.48	0.55	0.58	0.96
FG2	2	Fibroblasts	5	0.98	0.36	0.41	0.89	1.04	1.22	1.33
FG2	12	Fibroblasts	5	4.32	2.26	1.47	2.49	5.07	5.71	6.87
FG4	1	fibroblasts	5	0.79	0.28	0.43	0.56	0.91	0.97	1.08
FG4	2	Fibroblasts	5	0.90	0.25	0.59	0.70	0.91	1.13	1.16
FG4	12	Fibroblasts	5	1.33	0.88	0.68	0.74	1.11	1.30	2.83
MGA	1	Fibroblasts	5	1.20	0.59	0.77	0.79	0.83	1.48	2.12
MGA	2	Fibroblasts	5	1.68	1.12	0.65	0.93	1.17	2.29	3.34
MGA	12	Fibroblasts	5	4.49	1.42	2.20	4.13	5.03	5.33	5.78
MGA2	1	Fibroblasts	5	1.18	0.78	0.67	0.71	0.80	1.19	2.53
MGA2	2	Fibroblasts	5	2.42	1.95	0.96	1.47	1.57	2.28	5.81
MGA2	12	Fibroblasts	5	3.53	1.94	1.69	2.42	2.51	4.65	6.38
MGA4	1	Fibroblasts	5	1.61	1.23	0.74	1.09	1.22	1.22	3.78
MGA4	2	Fibroblasts	5	1.76	0.63	1.33	1.33	1.52	1.78	2.84
MGA4	12	Fibroblasts	5	2.91	2.16	1.32	1.36	1.72	3.89	6.27
E	1	Fibroblasts	5	1.66	1.64	0.53	0.81	0.94	1.50	4.53
E	2	Fibroblasts	5	5.69	2.58	2.48	4.58	5.13	6.91	9.34
E	12	Fibroblasts	5	6.55	1.36	4.84	6.10	6.43	6.80	8.59
E2	1	Fibroblasts	5	0.89	0.54	0.40	0.63	0.67	0.96	1.79
E2	2	Fibroblasts	5	2.54	2.62	1.04	1.25	1.55	1.65	7.21
E2	12	Fibroblasts	5	4.95	1.41	3.47	4.11	4.71	5.28	7.15
E4	1	Fibroblasts	5	0.78	0.41	0.38	0.53	0.59	0.99	1.38
E4	2	Fibroblasts	5	0.65	0.30	0.35	0.42	0.54	0.91	1.01
E4	12	Fibroblasts	5	4.83	0.97	3.29	4.75	4.90	5.31	5.92
MG	1	Inflammatory cells	5	0.04	0.02	0.00	0.04	0.04	0.05	0.05
MG	2	Inflammatory cells	5	0.06	0.03	0.02	0.04	0.06	0.06	0.09
MG	12	Inflammatory cells	5	0.27	0.17	0.10	0.11	0.25	0.38	0.50
FG	1	Inflammatory cells	5	0.00	0.00	0.00	0.00	0.00	0.00	0.00
FG	2	Inflammatory cells	5	0.01	0.02	0.00	0.00	0.00	0.03	0.04
FG	12	Inflammatory cells	5	0.22	0.18	0.05	0.06	0.20	0.29	0.49
FG2	1	Inflammatory cells	5	0.00	0.00	0.00	0.00	0.00	0.00	0.00
FG2	2	Inflammatory cells	5	0.01	0.02	0.00	0.00	0.00	0.00	0.05
FG2	12	Inflammatory cells	5	0.38	0.23	0.00	0.36	0.42	0.55	0.55
FG4	1	Inflammatory cells	5	0.01	0.01	0.00	0.00	0.00	0.00	0.02
FG4	2	Inflammatory cells	5	0.00	0.00	0.00	0.00	0.00	0.00	0.00
FG4	12	Inflammatory cells	5	0.05	0.09	0.00	0.00	0.00	0.05	0.22
MGA	1	Inflammatory cells	5	0.01	0.03	0.00	0.00	0.00	0.00	0.06
MGA	2	Inflammatory cells	5	0.09	0.07	0.00	0.04	0.07	0.14	0.18
MGA	12	Inflammatory cells	5	0.43	0.32	0.07	0.26	0.29	0.76	0.76
MGA2	1	Inflammatory cells	5	0.03	0.03	0.00	0.00	0.04	0.05	0.06
MGA2	2	Inflammatory cells	5	0.05	0.07	0.00	0.00	0.00	0.10	0.16
MGA2	12	Inflammatory cells	5	0.27	0.42	0.01	0.07	0.10	0.15	1.02
MGA4	1	Inflammatory cells	5	0.02	0.03	0.00	0.00	0.01	0.01	0.06
MGA4	2	Inflammatory cells	5	0.01	0.02	0.00	0.00	0.01	0.01	0.04
MGA4	12	Inflammatory cells	5	0.16	0.28	0.00	0.00	0.00	0.16	0.64
E	1	Inflammatory cells	5	0.10	0.06	0.04	0.05	0.10	0.11	0.18
E	2	Inflammatory cells	5	0.39	0.28	0.14	0.16	0.36	0.47	0.84
E	12	Inflammatory cells	5	0.33	0.13	0.19	0.20	0.36	0.43	0.47
E2	1	Inflammatory cells	5	0.21	0.07	0.10	0.18	0.25	0.25	0.28
E2	2	Inflammatory cells	5	0.10	0.14	0.00	0.02	0.05	0.10	0.34
E2	12	Inflammatory cells	5	0.25	0.10	0.14	0.18	0.22	0.32	0.36
E4	1	Inflammatory cells	5	0.08	0.10	0.01	0.04	0.05	0.05	0.25
E4	2	Inflammatory cells	5	0.06	0.05	0.00	0.01	0.05	0.09	0.13
E4	12	Inflammatory cells	5	0.16	0.06	0.08	0.12	0.16	0.21	0.23
MG	1	Connective tissue	5	6.46	5.26	2.27	2.34	3.51	10.76	13.45
MG	2	Connective tissue	5	10.88	4.58	2.98	11.51	11.80	14.05	14.06
MG	12	Connective tissue	5	40.25	13.43	24.43	33.88	35.59	48.75	58.59
FG	1	Connective tissue	5	1.40	0.70	0.78	1.03	1.26	1.36	2.59
FG	2	Connective tissue	5	2.92	0.94	1.45	2.74	2.99	3.61	3.83
FG	12	Connective tissue	3	14.00	6.10	6.99	6.99	16.91	18.10	18.10
FG2	1	Connective tissue	5	3.62	3.23	1.36	1.66	2.44	3.43	9.22
FG2	2	Connective tissue	5	2.12	1.00	1.11	1.39	1.78	2.87	3.44
FG2	12	Connective tissue	5	24.01	11.39	9.47	17.66	22.08	34.09	36.74
FG4	1	Connective tissue	5	3.91	2.16	1.54	2.61	3.03	5.83	6.56
FG4	2	Connective tissue	5	2.48	0.95	1.33	1.82	2.51	2.97	3.77
FG4	12	Connective tissue	5	8.87	2.27	5.33	8.59	9.18	9.70	11.57
MGA	1	Connective tissue	5	6.12	3.08	2.72	3.47	6.14	8.32	9.93
MGA	2	Connective tissue	5	4.43	2.34	1.77	2.22	4.97	6.35	6.86
MGA	12	Connective tissue	4	40.92	15.45	22.60	28.52	41.92	53.32	57.24
MGA2	1	Connective tissue	5	4.63	1.12	2.68	4.81	4.95	5.27	5.47
MGA2	2	Connective tissue	4	4.48	0.47	4.06	4.10	4.39	4.85	5.07
MGA2	12	Connective tissue	4	31.63	11.91	21.63	23.89	28.07	39.37	48.75
MGA4	1	Connective tissue	5	7.32	3.00	3.92	5.35	6.90	8.91	11.53
MGA4	2	Connective tissue	5	5.97	2.58	2.54	4.14	6.88	7.39	8.92
MGA4	12	Connective tissue	5	27.45	7.06	19.94	21.24	28.56	30.28	37.21
E	1	Connective tissue	5	9.76	7.77	3.79	4.91	5.06	13.07	21.98
E	2	Connective tissue	5	15.13	5.18	8.46	12.63	15.89	16.10	22.56
E	12	Connective tissue	5	27.54	9.75	15.24	20.77	28.41	33.75	39.52
E2	1	Connective tissue	5	6.39	1.50	4.57	5.28	6.79	6.92	8.41
E2	2	Connective tissue	5	11.22	11.71	5.32	5.60	5.75	7.31	32.11
E2	12	Connective tissue	5	30.79	7.71	21.26	28.30	28.82	33.37	42.20
E4	1	Connective tissue	5	3.73	0.95	2.32	3.30	3.98	4.30	4.75
E4	2	Connective tissue	5	7.35	2.45	3.24	7.07	8.25	8.81	9.39
E4	12	Connective tissue	5	35.07	3.42	32.04	32.10	33.92	37.73	39.58
MG	1	Blood vessels	5	0.00	0.00	0.00	0.00	0.00	0.00	0.00
MG	2	Blood vessels	5	0.20	0.40	0.00	0.00	0.00	0.00	1.00
MG	12	Blood vessels	4	12.00	2.80	8.00	10.00	13.00	14.00	14.00
FG	1	Blood vessels	5	0.00	0.00	0.00	0.00	0.00	0.00	0.00
FG	2	Blood vessels	5	0.00	0.00	0.00	0.00	0.00	0.00	0.00
FG	12	Blood vessels	3	5.00	1.00	4.00	4.00	5.00	6.00	6.00
FG2	1	Blood vessels	5	0.00	0.00	0.00	0.00	0.00	0.00	0.00
FG2	2	Blood vessels	5	0.00	0.00	0.00	0.00	0.00	0.00	0.00
FG2	12	Blood vessels	5	10.60	12.80	2.00	4.00	5.00	9.00	33.00
FG4	1	Blood vessels	5	0.00	0.00	0.00	0.00	0.00	0.00	0.00
FG4	2	Blood vessels	5	0.00	0.00	0.00	0.00	0.00	0.00	0.00
FG4	12	Blood vessels	5	0.00	0.00	0.00	0.00	0.00	0.00	0.00
MGA	1	Blood vessels	5	0.00	0.00	0.00	0.00	0.00	0.00	0.00
MGA	2	Blood vessels	5	0.00	0.00	0.00	0.00	0.00	0.00	0.00
MGA	12	Blood vessels	4	10.30	3.70	6.00	8.00	10.00	12.50	15.00
MGA2	1	Blood vessels	5	0.00	0.00	0.00	0.00	0.00	0.00	0.00
MGA2	2	Blood vessels	4	0.00	0.00	0.00	0.00	0.00	0.00	0.00
MGA2	12	Blood vessels	4	13.00	12.30	0.00	2.50	14.00	23.50	24.00
MGA4	1	Blood vessels	5	0.00	0.00	0.00	0.00	0.00	0.00	0.00
MGA4	2	Blood vessels	5	0.00	0.00	0.00	0.00	0.00	0.00	0.00
MGA4	12	Blood vessels	5	2.60	5.80	0.00	0.00	0.00	0.00	13.00
E	1	Blood vessels	5	0.20	0.40	0.00	0.00	0.00	0.00	1.00
E	2	Blood vessels	5	2.80	1.90	0.00	2.00	3.00	4.00	5.00
E	12	Blood vessels	5	9.80	1.50	8.00	9.00	10.00	10.00	12.00
E2	1	Blood vessels	5	0.00	0.00	0.00	0.00	0.00	0.00	0.00
E2	2	Blood vessels	5	1.00	1.40	0.00	0.00	0.00	2.00	3.00
E2	12	Blood vessels	5	3.40	1.50	1.00	3.00	4.00	4.00	5.00
E4	1	Blood vessels	5	0.00	0.00	0.00	0.00	0.00	0.00	0.00
E4	2	Blood vessels	5	0.00	0.00	0.00	0.00	0.00	0.00	0.00
E4	12	Blood vessels	5	3.00	1.20	2.00	2.00	3.00	3.00	5.00
MG	1	Residual substitute	5	46.06	11.17	28.32	42.68	50.00	52.41	56.88
MG	2	Residual substitute	5	32.50	8.96	19.98	26.56	35.40	40.25	40.31
MG	12	Residual substitute	5	10.46	4.02	3.41	11.45	11.74	12.21	13.51
FG	1	Residual substitute	5	12.55	3.75	9.17	10.69	10.75	13.49	18.65
FG	2	Residual substitute	5	15.96	7.49	9.72	11.22	11.34	20.64	26.90
FG	12	Residual substitute	3	19.34	3.33	15.67	15.67	20.16	22.18	22.18
FG2	1	Residual substitute	5	18.54	3.47	15.63	17.15	17.64	17.72	24.56
FG2	2	Residual substitute	5	19.77	3.26	16.12	17.62	18.84	22.41	23.85
FG2	12	Residual substitute	5	15.97	5.59	9.47	11.72	16.14	19.14	23.37
FG4	1	Residual substitute	5	25.96	3.47	22.78	22.91	24.89	28.85	30.35
FG4	2	Residual substitute	5	30.00	5.02	26.28	26.60	28.62	29.92	38.57
FG4	12	Residual substitute	5	29.09	1.70	27.68	27.76	28.12	30.90	31.00
MGA	1	Residual substitute	5	25.20	6.66	18.40	21.63	22.07	28.98	34.93
MGA	2	Residual substitute	5	22.17	4.26	16.12	19.48	24.03	24.69	26.52
MGA	12	Residual substitute	4	3.46	2.88	0.65	1.68	2.86	5.24	7.49
MGA2	1	Residual substitute	5	25.81	5.07	17.58	25.60	26.17	28.78	30.91
MGA2	2	Residual substitute	4	26.38	3.58	22.99	23.56	25.80	29.20	30.94
MGA2	12	Residual substitute	4	6.95	2.95	3.43	4.55	7.26	9.36	9.87
MGA4	1	Residual substitute	5	31.03	10.55	19.27	27.71	28.50	31.63	48.05
MGA4	2	Residual substitute	5	38.25	8.85	24.57	36.06	40.17	42.08	48.39
MGA4	12	Residual substitute	5	14.29	7.02	7.43	9.07	13.26	16.58	25.09
E	1	Residual substitute	5	n.a	n.a	n.a	n.a	n.a	n.a	n.a
E	2	Residual substitute	5	n.a	n.a	n.a	n.a	n.a	n.a	n.a
E	12	Residual substitute	5	n.a	n.a	n.a	n.a	n.a	n.a	n.a
E2	1	Residual substitute	5	n.a	n.a	n.a	n.a	n.a	n.a	n.a
E2	2	Residual substitute	5	n.a	n.a	n.a	n.a	n.a	n.a	n.a
E2	12	Residual substitute	5	n.a	n.a	n.a	n.a	n.a	n.a	n.a
E4	1	Residual substitute	5	n.a	n.a	n.a	n.a	n.a	n.a	n.a
E4	2	Residual substitute	5	n.a	n.a	n.a	n.a	n.a	n.a	n.a
E4	12	Residual substitute	5	n.a	n.a	n.a	n.a	n.a	n.a	n.a

*N* = number of specimens; *SD* = standard deviation; *Min* = minimum; *Q1* = first quartile; *Q3* = third quartile; *Max* = maximum; *MG* = collagen matrix (Geistlich Mucograft®); *FG* = volume-stable collagen matrix (Geistlich Fibro-Gide®) with twofold compression (FG2) and fourfold compression (FG4); *MGA* = volume-stable collagen matrix (creos.^tm^ mucogain) with twofold compression (MGA2) and fourfold compression (MGA4); *E* = scaffold made of polycaprolactone (PCL-12) with twofold compression (E2) and fourfold compression (E4)

## Data Availability

The data that support the findings of this study are available from the corresponding author upon reasonable request.
